# Aptamers Versus Vascular Endothelial Growth Factor (VEGF): A New Battle against Ovarian Cancer

**DOI:** 10.3390/ph16060849

**Published:** 2023-06-06

**Authors:** Yachana Mishra, Aditi Chattaraj, Vijay Mishra, Abhigyan Ranjan, Murtaza M. Tambuwala

**Affiliations:** 1 School of Bioengineering and Biosciences, Lovely Professional University, Phagwara 144411, Punjab, India; yachanamishra@gmail.com (Y.M.);; 2 School of Pharmaceutical Sciences, Lovely Professional University, Phagwara 144411, Punjab, India; 3 Lincoln Medical School, University of Lincoln, Brayford Pool, Lincoln LN6 7TS, UK

**Keywords:** ovarian cancer, vascular endothelial growth factor (VEGF), aptamer, antibodies, biomarker

## Abstract

Cancer is one of the diseases that causes a high mortality as it involves unregulated and abnormal cell growth proliferation that can manifest in any body region. One of the typical ovarian cancer symptoms is damage to the female reproductive system. The death rate can be reduced through early detection of the ovarian cancer. Promising probes that can detect ovarian cancer are suitable aptamers. Aptamers, i.e., so-called chemical antibodies, have a strong affinity for the target biomarker and can typically be identified starting from a random library of oligonucleotides. Compared with other probes, ovarian cancer targeting using aptamers has demonstrated superior detection effectiveness. Various aptamers have been selected to detect the ovarian tumor biomarker, vascular endothelial growth factor (VEGF). The present review highlights the development of particular aptamers that target VEGF and detect ovarian cancer at its earliest stages. The therapeutic efficacy of aptamers in ovarian cancer treatment is also discussed.

## 1. Introduction

According to a World Health Organization (WHO) report on cancer, there were about 10 million cancer-associated deaths in 2020. Almost one in six deaths is caused by cancer. Breast, lung, colon, and rectum cancers are the most prevalent in today’s society. The most common male malignancies are lung and prostate, while breast and ovarian cancers are often found in females [1,2,3,4,5]. Most patients’ cancer has metastasized by the time it is diagnosed. Modern techniques, including immunotherapy; RNA/DNA-based therapeutics; nano-drug delivery systems such as liposomes, carbon nanomaterials, and metallic nanoparticles; and other non-conventional approaches provide numerous opportunities for improved cancer diagnosis and therapy [6,7,8,9].

Ovarian cancer (OC) patients are mostly discovered at the late stage of metastatic illness. The limited treatment choices have resulted in OC being the leading cause of cancer-induced mortality among women [10]. Chemotherapy, radiation, and surgery have traditionally been the principal treatments for OC in prosperous nations [11,12,13]. Unfortunately, several issues plague these therapies, including frequent recurrence, drug resistance to several agents, subpar therapeutic effectiveness, and significant adverse effects. Although rapid developments in molecular biotechnology and chemical biology have significantly advanced the field of cancer theranostics, some hurdles still exist as cancer cells are inherently heterogeneous and complicated. Thus, the creation of high-performance detection methods for OC-related biomarkers such as vascular endothelial growth factor (VEGF) is urgently needed to accomplish early diagnosis and precise treatment [14].

VEGF has been identified as a key biomarker in the angiogenesis of numerous malignancies, including OC. Recent detection methods include surface plasmon resonance, colorimetric testing, fluorescence detection, and luminescence assays. These approaches, however, lack sensitivity and are time and money consuming. Thus, quick, easy-to-use, and highly accurate VEGF detection biosensors are essential [15,16,17].

Aptamers have a lot of potential for the creation of anti-VEGF treatments. These chemical antibodies are single-stranded oligonucleotide sequences that can bind to their targets through three-dimensional (3D) folding. They are screened by Systematic Evolution of Ligands by Exponential Enrichment (SELEX) technology for superior affinity, specificity, and targeting. Because of their distinct properties, such as simple synthesis, small size, easy modification, low molecular weight, low manufacturing cost, high thermal stability, convenient storage and transportation, low immunogenicity and toxicity, and lack of inter-batch variability, aptamers have a broad range of potential therapeutic applications [18]. Hence, by identifying the OC biomarker VEGF on the tumor cell surface and/or in the serum, new aptamers constitute intriguing platforms for precise OC detection [19].

In the present review, we summarize how aptamers have recently developed and been used to diagnose and treat OC by looking for the biomarker VEGF.

## 2. Correlation of Ovarian Cancer and VEGF

OC is the second most lethal and eighth most prevalent disease in women globally. OC affects about 300,000 women annually and is the cause of death of over 152,000 women among affected women, with an incidence of 3.4% and death of 4.7%, demonstrating the tremendous threat to women’s health and life. With a 30% survival rate, OC patients have a terrible prognosis [20]. Cytoreductive surgery and platinum-based chemotherapy are being used as the first-line treatment for OC [21]. Some patients may benefit from targeted therapy, including PARP inhibitors and anti-VEGF antibodies [22]. Nevertheless, the survival percentage will not increase much because more than half of patients will suffer a recurrence within two years [23].

Early-stage illness has a roughly 92% five-year overall survival rate compared with 29% for late-stage disease. More than 70% of patients receive an innovative diagnosis because of the absence of typical symptoms and signs in the initial stages of cancer and the aggressive tendency of OC to move from an early to an advanced stage in less than a year. An early identification and detection are thus crucial to enhancing the prognosis. A serum biomarker is a practical, affordable, and non-invasive way to diagnose cancer, and research has been done to find biomarkers that are more trustworthy for OC early detection [24].

### 2.1. VEGF: Biomarker of Ovarian Cancer

**Figure 1:**
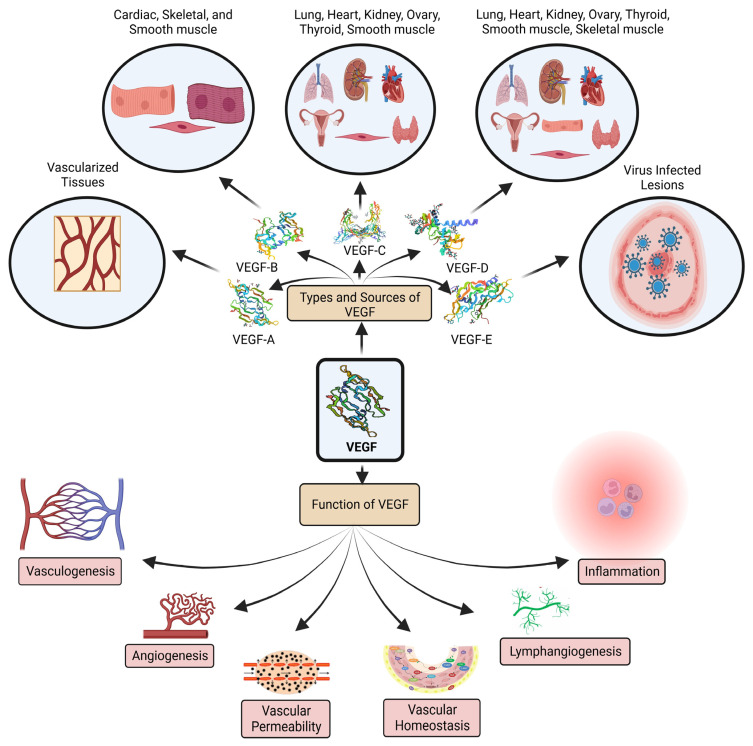
VEGF family members, primary biological sources, and functions.

One of the most prevalent and significant angiogenic routes in OC is the VEGF/VEGF receptor (VEGFR) pathway. OC cells produce VEGF and VEGFR. The overexpression of VEGF indicates a deprived prognosis [25]. The most significant pro-angiogenic factor is probably VEGF. Two placental growth factors and five glycoproteins, namey VEGF-A, B, C, D, and E, comprise the VEGF-associated gene family for angiogenesis (PLGF-1 and -2) (Figure 1). Vascular permeability factor VEGF-A is essential for both healthy and unhealthy angiogenesis. In addition to tumor cells, perivascular cells can also generate VEGF. A paracrine mechanism affects endothelial cells to encourage vascular development, prevent endothelial cell death, and increase vascular permeability. According to observational studies, VEGF is overexpressed in most human malignancies and is directly linked to the progression, metastasis, pathological grade, and poor prognosis of ovarian cancer. As a result, VEGF is frequently utilized as a circulating marker to identify the emergence and progression of tumors. In the early stages of carcinogenesis, VEGF facilitated the transition to the angiogenic phenotype. A sign of a malignant process is thought to be when tumor cells change into an angiogenic phenotype [26].

The most accessible VEGF isoforms to date have been found, and they exist as highly soluble molecules. VEGF-A can bind to both VEGFR-1 and 2; however, VEGF111 binds to VEGFR-2, and VEGF111 affects VEGFR-2 while forming the complex NRP-1/VEGFR-2 by insufficiently attaching to the co-receptor neuropilin-1 (NRP-1) explained through the protein kinase C (PKC)-extracellular signal-regulated kinase (ERK) 1/2 pathway. In OC, VEGF111a is highly expressed [27,28].

### 2.2. VEGF: Targeted Conventional Treatments and Their Limitations

Considering the symptoms are often mistaken for those of other prevalent illnesses, most OC patients only receive a late diagnosis, which has a dismal prognosis. The majority of OC patients are expected to have a recurrence and eventually develop chemo-resistant cancer, despite initially responding to cytoreductive surgery as well as carbo-taxol-based treatment. Additional chemotherapy rounds are administered to individuals with recurring diseases, but these treatments ultimately do not cure them. A continually growing number of tumors have been successfully treated with tumor immunotherapies, including checkpoint inhibitors, although only around 10% of patients with OC have shown unbiased responses in clinical studies [29,30].

For more recent immunotherapeutic approaches to OC, understanding immune activation pathways and immune suppressive mechanisms in the tumor microenvironment (TME) is essential. T cells and natural killer (NK) cells, tumor-associated neutrophils (TAns), tumor-associated macrophages (TAMs), and myeloid-derived suppressor cells (MDSCs) are a few examples of both innate and adaptive immune cells, which shape the peritoneal TME directly or indirectly (via soluble interactions), fostering a favorable environment for the growth of tumors and a metastatic soil [31].

The development of a sophisticated immune suppression network that efficiently neutralizes the anticancer activity of TAMs, TANs, γδT cells, and NK cells is one of the primary causes of disease progression and therapy failure. To make matters worse, by upregulating VEGF and matrix metalloproteinase (MMP) production, TAMs, TANs, and NK cells develop pro-tumor activities supporting tumor vascularization metastasis [32].

According to recent data, conventional therapy may partially restore the balance between immune surveillance and tumor development, two important components of tumor escape. Primary cytoreduction has been linked to both a quantitative and qualitative rise in CD8 T-cells and a considerable reduction in circulating T lymphocytes with an immunosuppressive function (T-regulatory cells). Recent research has revealed that the vital balance between immune-activating and immune-suppressing processes is lost when oncogenesis and cancer development occur. OC creates a highly suppressive environment to evade the immune system. Ovarian tumor tissue contains immune-activated tumor-infiltrating lymphocytes (TILs), which are evidence that the immune system is the neoplasm’s trigger. It has been shown that the TIL milieu is linked to improved prognosis and increased chemosensitivity, with a higher rate of optimum residual tumors after the initial cytoreduction. In order to select patients for clinical trials as efficiently as possible and to better understand how these tumor biomarkers relate to immunotherapeutic responses, scientists are currently concentrating their attention on novel immunologically effective tumor biomarkers [33].

#### 2.2.1. Anti-VEGF Antibodies

There are VEGF isoform-specific antibodies. The VEGF family comprises five proteins, i.e., VEGF-A, VEGF-B, VEGF-C, VEGF-D, and VEGF-E. Various agonistic VEGF isoforms bind to different VEGF receptors [34].

Antibodies against VEGF-A malignant tumors can now be treated with bevacizumab [35]. A bispecific antibody called Faricimab (also known as Faricimab-svoa; Vabysmo^TM^) binds to and inhibits both VEGF-A and angiopoietin-2 (Ang-2) [36].

In addition to controlling glycolipid metabolism and decreasing the buildup of inflammation and reactive oxygen species (ROS), a combination of a VEGF-B monoclonal antibody and interleukin-22 (IL-22) may protect against diabetic neuropathy [37]. The emergence of cancers is decreased by the recombinant fusion protein Aflibercept, which binds with the placental growth factor VEGF-A and VEGF-B [38].

A phage-derived human monoclonal antibody against VEGF-C was created as a possible tumor treatment. It cleared quickly in preclinical animals, with serum clearing noticeably quicker than plasma. It was discovered that the antibody formed a complex with VEGF-C after radiolabeled anti-VEGF-C was incubated in animal and human blood, plasma, or serum [39]. Antibodies against 1E9 prevented endothelial cells activated by VEGF-C from proliferating and migrating by inhibiting the activation of VEGFR3 signaling. Additionally, they stopped the development of renal cancer cells that express VEGF-C by inhibiting NRP2 signaling [40]. VEGFR2 is the target of the fully human antibody KD035, which prevented the activation of VEGFR2 by VEGF-A and VEGF-C [41].

Ramucirumab, a monoclonal anti-VEGFR2 antibody, prevents endothelial cell proliferation and VEGF-A, VEGF-C, and VEGF-D binding [42].

Many VEGF inhibitors have received approval for cancer treatment during the past decades. The humanized monoclonal immunoglobulin G1 (IgG1) antibody bevacizumab has demonstrated positive effects in the treatment of lung cancer by binding to VEGF-A. Studies have shown that bevacizumab added to chemotherapy increases overall survival (OS) and progression-free survival (PFS) in lung tumor patients compared with chemotherapy alone. Bevacizumab has also shown positive results when used in chemoimmunotherapy. It can prolong the survival rate [43,44].

Bevacizumab’s safety has been in question due to issues with wound disruption, hypertension, venous or arterial thrombosis, and gastrointestinal perforation or fistula. The problem with bevacizumab for advanced OC in the high-risk subgroup is its high cost. Bevacizumab needs to be priced between 46% and 67% less to be cost-effective in a high-risk subpopulation. Bevacizumab therapy has been linked to a decline in quality of life, and there are still no reliable indicators for predicting the survival advantages of the drug [45,46]. Bevacizumab is taken for 15 months in primary OC; however, its benefit on PFS vanishes after two years and does not increase OS [47].

#### 2.2.2. Immune Checkpoint Inhibitors

The expression levels of VEGFA and HIF-1 are significantly correlated with the programmed cell death protein (PD-L1) expression levels. By influencing different immune cells in TME, VEGF-A promotes immunosuppression and reduces immune activation. Hence, treating cancer patients with a combination of VEGF and immune checkpoint inhibitors (ICI) can reduce immunological escape. Three primary kinds of ICI, namely anti-PD-L1, anti-PD-1, and anti-CTLA-4, are now recognized as first-line treatments. Pembrolizumab, nivolumab, toripalimab, sintilimab, atezolizumab, durvalumab, and ipilimumab are the principal inhibitors utilized for first-line cancer therapy [48]. ICI can sometimes prevent cancer patients’ diseases from progressing, while in other cases, they provide no further advantages [49]. ICIs differ from other anticancer medication classes in several ways. In particular, they produce unfavorable immune-related adverse events irAEs, which are inflammatory side effects. Hyperactivation of the immune system affecting the gastrointestinal tract, endocrine organs, and skin may result in irAEs. Typical irAEs include colitis, pneumonitis, skin rash, hypophysitis, and hepatitis. Some individuals experience irAEs that resemble autoimmune conditions such as polymyalgia-like syndromes, myositis, and arthritis [50].

#### 2.2.3. Tyrosine Kinase Inhibitors

By inhibiting and down streaming VEGFR signals, tyrosine kinase inhibitors (TKIs) can selectively induce podocytopathy, suppressing endothelial growth and maintenance. Increased c-mip expression and decreased RelA expression in the podocytes of TKI-receiving patients suggest that the possible mediators of this process are RelA and c-mip intracellular proteins. Patients receiving TKI treatment also experienced TMA-like lesions in addition to podocytopathy. Ibrutinib, an inhibitor of Bruton tyrosine kinase (BTK), has recently been linked to renal damage. BTK generates cytokines, inflammatory mediators, and immune system regulation. Ibrutinib has thus been used to treat individuals with mantle cell lymphoma and chronic lymphocytic leukemia [51].

## 3. Aptamers

**Figure 2:**
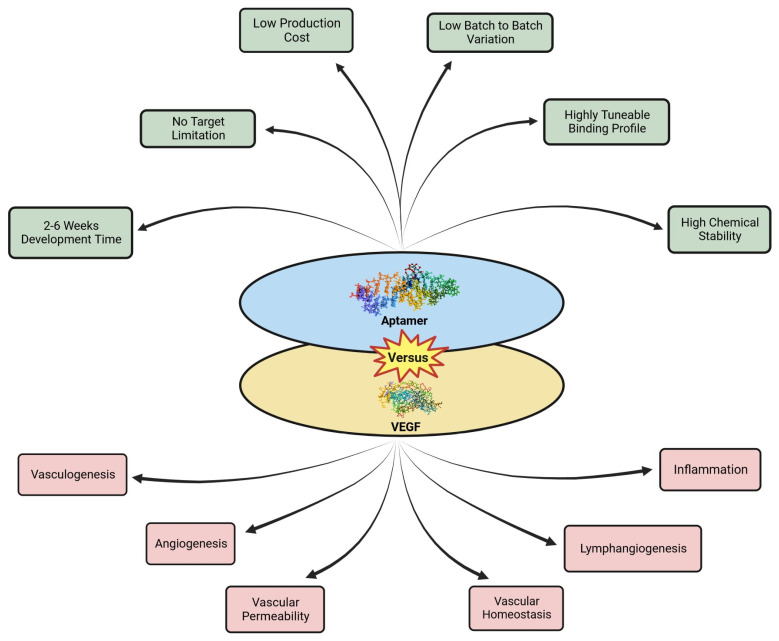
Aptamer versus VEGF.

Peptide or oligonucleotide aptamers are one of the most recently produced molecular therapies (Figure 2). Two different teams, Tuerk and Gold, and Ellington and Szostak, extracted oligonucleotide sequences for selectively attaching various substrate molecules and made the initial discovery of aptamers and developed a novel, very general, and versatile, methodology, i.e., SELEX, in the early 1990s. The subset of RNA molecules, SELEX, was isolated from a population of random sequence RNA molecules and binds precisely to a range of organic dyes. One in ten RNA molecules with random sequences folds in a way that creates a particular binding site for small ligands. The aptus, a Latin word that means to fit, and meros, a Greek word that means part, are the origins of the term aptamer [52].

When aptamers engage with the chosen targets, they exhibit a remarkable selectivity, mimicking antibodies. The discovery of isolated oligonucleotide molecules bound by particular ligands was the first proof of the aptamer binding capacity. These oligonucleotide aptamers were produced by the in vitro SELEX method. A short sequence of RNA or ssDNA (20–100 bases long) has a three-dimensional structure that enables aptamers to engage with specific ligands with an affinity and selectivity comparable with antibodies. Nonetheless, aptamers are preferable to antibodies due to their quick and inexpensive manufacturing. A study showed that creating a 5 to 20 amino acid chain and encasing it in a peptide scaffold produced a specific protein. A peptide aptamer is a novel idea introduced for this kind of aptamer. Oligonucleotide and peptide aptamers have been created for various therapeutic and diagnostic purposes. Aptamers have been used for the prognosis, diagnosis, and treatment of several diseases, including cancer [53].

### 3.1. Synthesis of Aptamers

A DNA pool containing around 10^14^–10^17^ distinct oligonucleotide molecules is first chemically synthesized following the concept of aptamer manufacturing using SELEX. Screening out and amplifying these particular oligonucleotides is the purpose of the subsequent step, which often needs multiple amplification and selection cycles. The affinity of chosen oligonucleotides to the target increasingly rises as the selection procedure progresses and the selection requirements are more stringent. Additionally, point mutations may occur due to inaccurate PCR amplifications, leading to the new oligonucleotide generation. Furthermore, the oligonucleotide’s specificity and binding affinity may be improved during selection. The final DNA or RNA oligonucleotides produced are the ideal aptamers for the target because they attach to the target extremely precisely and firmly [54].

#### 3.1.1. Capillary Electrophoresis SELEX

The capillary electrophoresis SELEX (CE-SELEX) approach has been improved by separating unbound and bound sequences based on electrophoretic mobility variations. The number of selection rounds is decreased due to the high-resolution separation. Yet, the target size affects the resolution. The precise electrophoretic mobility shift followed by complex formation is only simple to obtain for giant target molecules (such as proteins). The library size is often lowered to 1012 sequences because of the tiny injection quantity used in CE, and/or more significant target and aptamer concentrations are needed, which might result in non-specific interactions. When CE-SELEX is run via a micro-free-flow electrophoresis device (μFFE), considerable improvements are shown. The time required for fraction collection is reduced with the use of μFFE. After only one selection round, low nanomolar affinity sequences were discovered, suggesting that aptamers may be created even more quickly [55].

#### 3.1.2. Capture SELEX

This method is used to prevent target immobility. Stoltenburg et al. initially created it for the selection of the structure-switching signaling aptamers. A randomized ssDNA oligonucleotide library is the first step in the capture-SELEX method. This library is built with a complementary docking sequence to capture the library on a solid substrate. The library is incubated with an aliquot of magnetic beads that have been treated with capture oligos so that there is a hybridization between the capture oligos and the docking sequence of the original library on the magnetic beads, causing an immobilized library. Moreover, the immobilized ssDNA library and the target molecule are incubated together. DNA-bead complexes release the oligonucleotides with an affinity towards the target molecule, which then fold into the target’s 3D structure in a solution. For the next selection phase, the supernatant is magnetically separated from the sample and amplified by PCR [55,56].

#### 3.1.3. Magnetic-Bead-SELEX

In the magnetic-bead-SELEX process, the DNA pool and magnetic beads are incubated, and as a result, the DNA pool is chemically altered from the chemical interaction with the target molecule. The target-bound sequences are removed from the targeted magnetic beads combined with denaturing or heat treatment after magnetically separating unbound oligonucleotides. Using certain primers, the chosen oligonucleotides are amplified using PCR. The pertinent sequences utilized in the following SELEX cycle are further purified. In recent work, six novel aptamers for lung tumor biomarkers were chosen to utilize serum and magnetic-bead-SELEX from lung tumor patients. The modeling of the secondary structure of these aptamers and the binding affinity characterization were also presented [57,58,59].

#### 3.1.4. Cell-SELEX Method

Positive and negative selections are used in the cell-SELEX approach for target and non-target cells, respectively. This technique results in the identification of biomarkers and aptamers, which are helpful in diagnostic and therapeutic applications. Only cell surface chemicals are affected. Consequently, healthy cells are successfully selected. Fluorescence-activated cell sorting (FACS) and magnetic bead separation are methods developed to improve specific binding. In 2016, a novel technique known as ligand guided selection (LIGS) was announced to obtain very precise aptamers against cell surface receptors on a particular cell. Williams et al. developed LIGS, a version of SELEX, to find extremely precise aptamers against intricate cell-surface markers in their natural state. A multiplexed ligand discovery platform that uses SELEX-targeting membrane receptors in its initial functional state may be created by extending LIGS to detect two receptors on the same cell surface [60]. Using LIGS, Freage et al. identified a group of aptamers able to selectively recognize the TCR-CD3 complex produced in human-cultured T lymphocytes and T cells collected from healthy persons [61]. Recently, utilizing the same technique, Moccia et al. discovered two brand new, membrane-bound IgM (mIgM)-specific truncated G-rich aptamers with the names R1.2 and R1.3 [62]. A competitive elution with a monoclonal antibody selects an explicit target on the cell surface [63].

#### 3.1.5. Post-SELEX Modifications

A range of post-SELEX chemical modifications for selected aptamers have been proposed to prevent exo- or endo-nucleases from degrading DNA or RNA aptamers, to prolong their half-life in vivo, or even increase their binding affinity. Compared with DNA aptamers, RNA aptamers have a more composite 3D structure, with a single chain structure and the presence of an extra hydroxyl (OH) group at the 2′ position of the ribose sugar. Unfortunately, the utility of RNA aptamers is severely limited by cleavage by common RNases. The chemical modification of RNA aptamers at the 2′ position of ribose sugar with -OCH_3_, -NH_2,_ and -F helps in overcoming their resistance toward nucleases. The vulnerability of aptamers to renal filtration is also increased because their size is smaller than that of antibodies. RNA modified at 2′ amino or 2′ fluoro nucleotides at each of the cytidine and uridine sites was found to be protected from degradation by 10^3^ times plasma, which is 5 to 15 h compared with the unmodified RNA in rabbit serum [64].

The modified post-SELEX method allows non-canonical ribonucleotides to be integrated during in vitro transcription. Consequently, it was shown that a T7 RNA polymerase mutant could easily transcribe an RNA library made up of high hydrophobic residues, known as fGmH RNA. This led to the choice of the *Staphylococcus aureus* Protein A (SpA) aptamer. It is also feasible to modify the bases, often by adding new chemical moieties to the pyrimidines’ position 5 atom. This method is used in a commercially available technique to produce the slow off-rate modified aptamer (SOMAmer). Lastly, Spiegelmers can exhibit strong resistance to nuclease destruction, while preserving their affinities to bind [65].

### 3.2. Properties of Aptamers

Aptamers have several unique characteristics, including a high selectivity and affinity when binding targets. In addition to directly altering target molecules, aptamers may combine with and transport other medicines into cells. Aptamers can be produced against severe biotoxins as they do not require animal vaccination during their manufacture. They can also be grown against non-immunogenic compounds, including tiny chemical and inorganic molecules and metal ions. Their production takes less time because aptamers are created in vitro rather than through extensive animal vaccination. Simple modifications may be made to expand the uses and stability of aptamers. With RNA aptamers, stability is particularly crucial as, when utilized in vivo, RNA aptamers are susceptible to nuclease destruction. A slight variance occurs from batch to batch with aptamers. Making aptamers is less expensive than creating mAbs. Alternatively, aptamers may always be chemically produced and PCR amplified once their sequence has been determined. Aptamers are not temperature sensitive; they can withstand temperatures of 80 °C. Despite the fact that they have been denatured, few aptamers immediately return to their former structure. Numerous aptamers can adopt kinetic configurations that do not reform after denaturation. When employed for therapeutic applications, aptamers of 20,000 Da may easily permeate tissues such as malignancies [66].

### 3.3. Classification of Aptamers

#### 3.3.1. RNA Aptamers

Electrostatic interactions, van der Waals forces, and hydrogen bonds modify the recognition abilities of specific ions, proteins, cells, and polymers by altering the tertiary or secondary structures [67]. As a result, aptamers in the realm of theranostics have developed into intelligent, targeted, and high-affinity probes with various medicinal applications by forming cross-linkers and bioactive groups. RNA-based aptamers such as short hairpin RNA (shRNA), small interfering RNA (siRNA), and microRNA (miRNA) are used in clinical settings. They are thought to be cutting-edge treatments for malignant tumors because of their targeted cytotoxic effects on in vivo and in vitro cancer cell lines [68].

#### 3.3.2. DNA Aptamers

A DNA aptamer is a short and high-affinity oligonucleotide. It may fold into a particular form because of its single-stranded nature (secondary or tertiary). Many weak interactions, such as van der Waals forces, hydrogen bonds, electrostatic interactions, and Π stacking, work together to enable aptamer binding. The ability of an aptamer to connect to different targets, ranging from a single metal ion to a giant cell, depends on its unique structure and binding forces. Regarding point-of-care diagnostics, the single-stranded DNA (ssDNA) aptamer provides several benefits for early diagnosis. Compared to antibodies, aptamers have a low molecular weight and excellent stability. They may also be produced at a low cost and with ease on a large scale. As a result, they can serve as capture probes in place of antibodies. Using a screening procedure known as SELEX, an aptamer that best binds with a target is chosen from a pool of 10^15^ random library sequences. The most precise and highly-affine aptamers are screened by successive binding, washing, elution, and amplification in SELEX procedures [69]. The distinctions between RNA and DNA aptamers are as follows:

Single-stranded RNA molecules called RNA-aptamers are naturally water-soluble and cannot be produced sufficiently to modify protein activity effectively. Compared with RNA aptamers, native DNA aptamers are more stable. A DNA aptamer has an in vitro half-life of around 30 to 60 min, while an RNA aptamer only has a few seconds. Single-stranded DNA oligonucleotides make up the beginning library for selecting DNA aptamers. A double-stranded DNA library is translated to produce the first RNA library for RNA aptamer selection. To simplify DNA PCR with consequent transcription for the following round of SELEX, reverse transcription for RNA aptamers must be carried out in each selection round. Compared with DNA aptamers, RNA aptamers generate more varied and complex 3D structures, providing a wider range of conformations [70].

#### 3.3.3. Peptide Aptamers

Recent advancements in the realm of aptamers include peptide aptamers. These are flexible, “doubly inhibited”, unlike their nucleic acid-based counterparts. Aptamers made of peptides have a variety of crucial functional groups that DNA/RNA lack. Bonding properties provide the peptide aptamers with special target affinity and interaction abilities. The creation of libraries is followed by the amino acid sequence amplification and isolation step using a high-affinity target that is then eluted. A strict and rationalistic selection technique makes the peptide aptamer resistant to DNAse- and RNAse-mediated lysis by considering the scaffold selection, peptide length, and marker count [71].

#### 3.3.4. DNAzyme-Assisted Aptasensors

An aptamer and the catalytically active nucleic acid unit, either ribozyme or deoxyribozyme, are created as part of DNAzyme-assisted aptasensors. The full aptazyme undergoes significant physical changes due to aptamer-target binding. Thus, aptazymes work similar to allosteric enzymes. The binding of effectors (ligands) to the allosteric sites regulates its catalytic action. Aptazymes contain an aptamer at their allosteric location. Aptazymes may be produced for various uses and have already been used to regulate gene expression and conduct analytical testing [72,73].

### 3.4. Aptamers in Ovarian Cancer Treatment

In a study, Heidari et al. used the hydrothermal approach to create green carbon dots (CDs) from lemon extract, which were then coupled to capture probes and aptamers linked to the cancer antigen-125 (CA-125) OC biomarker using covalent and hybridization tests, respectively. For the CD-probe−apt conjugate, four DNA aptamers were used. All conjugates were evaluated for their ability to identify OVCAR-3 cells that were CA-125 positive. The evaluation of CD-probe and CD-probe−aptamer synthesis was supported by an increase in surface roughness as determined by AFM analysis, a decrease in CD fluorescence intensity following bioconjugation, and an increase in the construct particle size. Cellular imaging employed conjugates with a low cytotoxicity, suitable zeta potential, and effective aptamer release. This focused diagnostic procedure used the four published DNA aptamers to increase fluorescence. This work established that using CD-probe−aptamer conjugates as non-toxic agents can create new opportunities for fluorescence nano-imaging in diagnosing specific cancer cells [73].

#### 3.4.1. Detection of Human Epididymis Protein 4

Hanžek et al. developed the oligonucleotide diagnostic probe aptamer to identify the human epididymis protein 4 (HE4) OC biomarker in urine. In contrast with healthy or benign circumstances, OC exhibits an overexpression of the protein HE4. Urine HE4 is a promising non-invasive biomarker for OC screening due to its excellent stability and diagnostic usefulness. Bioinformatics analysis and DNA sequencing are used to find the anti-HE4 aptamers. The dissociation constant (K_d_) of the (AHE3) and K_d_ of the (AHE1) aptamers are 127 ± 28 nM and 87 ± 9 nM, respectively. AHE3 and AHE1 are anti-HE4 aptamers, which bind to HE4 proteins in urine. Hence, these aptamers may be useful diagnostic tools for developing biosensors or urine tests for OC [74].

#### 3.4.2. Heat Shock Protein 70 Detection

Tx-01, a brand new aptamer, can detect serous carcinoma cells and tissues precisely. Lin and his research group aimed to define Tx-01′s clinical function and potential molecular processes in OC. Immunostaining with statistical analysis was used to determine the interaction between the intracellular domain of heat shock protein 70/Notch1 (HSP70/NICD) and Tx-01 in OC. Experiments were conducted to illustrate the probable mechanisms of Tx-01 in vitro and in vivo. Tx-01 inhibited the nuclear translocation of HSP70 by preventing the intracellular HSP70/NICD junction and reducing the migration and invasion of OC OVCAR3 serous cells. Tx-01 also slowed the in vivo development of serous-type OVCAR3 cell tumors. Tx-01 interacts with membrane-bound HSP70 (mHSP70 and does not directly interact with NICD) in circulating ascites tumor cells (CTCs) to function as a prognostic factor. It is also believed to be important for the activation and recognition of NK cells. Tx-01 improved the prognosis and provided therapeutic advantages in serous OC by interacting with HSP70. Tx-01 could work as an effective inhibitor for the treatment of serous OC [75].

#### 3.4.3. Molecular Therapy

Wang et al. used cell SELEX to target patient-derived primary serous OC (pSOC) cells. A DNA aptamer called mAPoc46 was introduced. By using flow cytometry, an average K_d_ of 0.15 ± 0.05 M was discovered. The pSOC cells were very selective to the mApoc46 aptamer. After 30 min, FAM-labeled mApoc46 stained only the live pSOC cells. Notably, FAM-mApoc46 showed excellent selectivity against low-grade SOC, borderline ovarian tumors, other non-epithelial OC, and healthy ovarian tissue against high-grade serous OC tissue (HG-SOC) in cryosections. These findings suggest a possible use for identifying the histological subtypes of OCs while operating. Cy5-tagged mAPoc46 is concentrated on tumor sites and acts as an in vivo imaging probe in a patient-derived tumor xenograft NCG mouse model. The mApoc46 probe demonstrated a reliable and consistent ability to detect SOC [76].

#### 3.4.4. Aptamer Functionalized Liposome

Non-canonical nucleic acid structures called G-quadruplexes are created by stacking guanine tetrads. A 26-mer G-rich DNA oligonucleotide called AS1411 may form a variety of G-quadruplexes. It has been shown to have a cancer-specific antiproliferative effect. It was later discovered to be an aptamer to nucleolin. This multi-functional protein preferentially binds quadruplex nucleic acids and is abundantly present on the surface of the cancer cell. Comparing AS1411 to non-quadruplex DNA sequences, cellular internalization is particularly effective [77]. In addition to promoting the compliance of basal cell carcinoma patients, topical liposomal medication formulations comprising AS1411-aptamer-attached liposomes were intended to deliver 5-fluorouracil to the tumor site in a sustained manner [78]. Its potential utility as a theranostic nanoprobe for renal cancer was demonstrated by the introduction of the AS1411 aptamer, which further improved the MRI effect and the tumor growth inhibitory effect [79]. The nucleolipidic Ru(iii) complex HoThyRu, chosen as an anticancer drug, as well as the nucleolin-targeting AS1411 aptamer, permitting the selective identification of cancer cells, were the two active components that Riccardi et al. explored loaded with niosomes considering their physicochemical and biological features. The bioactivity of the niosomes containing Ru(iii) was significantly enhanced by AS1411 in each of the evaluated cell lines [80].

Jiang et al. created nanosized AS1411, an aptamer-functionalized liposome. In cancer cells, lipofectamine-based miR-29b demonstrated a usual concentration-dependent lethal impact. LP-miR significantly decreased A2780 cells’ vitality compared with the untreated control, but was unaffected by LP-Mut (mutant loading), highlighting the significance of precise gene sequencing. Green fluorescence was significantly reduced by LP-miR, indicating that the cell viability had decreased. In addition, more PI-positive cells were seen in the live/dead assay. The LP-miR-treated cells showed intense fluorescence, which indicated the presence of apoptotic cells. This unique aptamer-directed liposome loaded with miR-29b may offer a novel platform for improving the treatment result of ovarian malignancies [81].

#### 3.4.5. Aptamer-Magnetic Mesoporous Silica Nanoparticles

Magnetic mesoporous silica nanoparticles (MMSNPs) are crucial components of targeted treatment. Torabi et al. created magnetic cores utilizing the thermal breakdown technique. MMSNPs were loaded with sunitinib (SUN), and the aptamer for mucin 1 (MUC-1) was modified with an amine. Compared with MUC-1-negative MDA-MB-231 cells, in vitro biological studies showed that cells overexpressing MUC-1 (OVCAR-3) had the most significant effect on MMSNP targeting. MMSNP-SUN-MUC-1 was developed as a special multi-functional targeted delivery carrier against the overexpression of MUC-1 in OC cells [82].

#### 3.4.6. Aptasensors

OC is one of several malignancies that overexpress the glycoprotein carcinoembryonic antigen (CEA). As CEA is a significant tumor biomarker, its quantification aids in cancer diagnosis, tracking tumor development, and subsequent therapy. Ma et al. created a very sensitive sandwich aptasensor for CEA detection on sensors with interdigital electrodes. A chemical linker binds a larger anti-CEA capture aptamer of a carbon-based substance to the sensor surface, and then the aptamer measures the CEA. Additionally, immobilized aptamers were used in the CEA blood tests, which did not alter the target validation. The CEA detection limit in PBS and serum was determined to be 0.5 ng/mL [83].

The work of Farzin et al. reported a high-performance biosensing nano platform AgNPs-PAN-oxime NF composed of amidoxime-modified polyacrylonitrile nanofibers decorated with silver nanoparticles. The developed aptasensor showed considerable selectivity, reproducibility, and stability. Compared with the ELISA technique, the favorable result of the CA 125 measurement in the blood samples suggests that the aptasensor can be used clinically to monitor tumor biomarkers for the early detection and treatment of OC [84]. OC has several biomarkers and aptamers can detect them efficiently (Table 1).

### 3.5. Toxicological Profile of Aptamers

#### 3.5.1. Aptamer-Based Targeted Chemotherapy

A unique chemically altered nucleic acid aptamer with a length of 35 nucleotides containing a stem−loop motif that is particular to the T24 bladder cancer cell line was discovered by Wang et al. In mouse orthotopic heterograft models of human bladder tumors, the B1-nanotrain-epirubicin construct outperformed epirubicin and demonstrated specific cytotoxicity towards bladder cancer cells. Targeted chemotherapy for bladder cancer is made achievable by this aptamer-based delivery system, offering a strong justification for clinical development [86].

Wang et al. created a delivery vehicle for actively targeted medication based on the AS1411 aptamer, a 26 bp single-stranded DNA oligonucleotide, enhancing the HAPs’ enrichment in the 4T1 tumor cell line. The TPZ@Apt-MOF (TA-MOF) drug delivery system combines the hypoxia-activated prodrug TPZ and surface-engineered nucleolin aptamer AS1411. AS1411-engineered MOF has demonstrated more potent in vivo and in vitro tumor targeting effects than naked MOF. GSH causes MOF to break down inside the tumor, generating Fe^2+^ and releasing the cargo. The tumor-protecting agent GSH is heavily consumed during this process. Then, the superoxide anions produced inside cells due to the Fenton reaction, which Fe^2+^ mediates, are created at a higher rate [87].

#### 3.5.2. In Vivo and In Vitro Cytotoxicity

With carriers, including antibodies, peptides, and aptamers, targeted treatment may transfer medications precisely to cancer cells. Chen et al. developed a complex conjugate of aptamer and cyclometalated iridium (III) (ApIrC) as a focused anticancer drug. Compared with the iridium complex alone, ApIrC significantly increased cellular absorption in cancer cells while showing a beneficial lower toxicity than normal cells. ApIrC can preferentially concentrate in the mitochondria after being taken up by cells through endocytosis and can cause caspase-3/7-dependent cell death. Surprisingly, ApIrC has a high tumor permeability and can target 3D multicellular spheroids (MCSs) preferentially. As a result, it can efficiently harm cells inside MCSs [88].

For the treatment of solid malignancies, Qian et al. developed an aptamer−M1 macrophage (ApEn-M1) conjugate that demonstrated improved targeting capacity in vivo and in vitro in tumor cells, leading to a notable cytotoxicity. In addition, in a mouse model of pulmonary metastasis from a breast tumor and breast tumor xenograft, ApEn-M1 demonstrated a higher antitumor effectiveness. Interestingly, ApEn-M1 might boost T cell infiltration and activity to change the immune microenvironment [89].

Yavari et al. created aptamer-activated nanoparticles (AP-NPs) that target the epithelial cell adhesion molecule (EpCAM) to improve therapy effectiveness for colorectal cancer (CRC). By administering the CT-26 cell line subcutaneously, tumor-bearing BALB/c mice were developed for in vivo investigation. The findings showed that synthesized AP-FU-NPs had a spherical surface with a size of 101 nm, were reasonably homogenous, and had a reasonable encapsulation effectiveness (83.93%). AP-FU-NPs were much less harmful to HEK-293 cells, according to the in vitro tests, but they were significantly more toxic against HCT-116 cells and CT-26. Moreover, in vivo findings have not revealed an appreciable hemolytic impact, weight loss, or liver or kidney damage [90].

Zhu et al. developed aptamer-decorated daunorubicin-containing NPs (AP-Drn NPs) and transferrin-decorated luteolin-loaded NPs (Tf-Lut NPs). They hybridized them to form AP/Tf-Drn/Lut NPs. The system’s in vitro and in vivo effectiveness was assessed using leukemia cell lines and a cancer-cell-carrying mouse model. AP/Tf-Drn/Lut NPs showed noticeably more cytotoxicity outside the body. As a result of the two medications working synergistically, AP/Tf-Drn/Lut NPs demonstrated more inhibition to the tumor cells. The most potent anti-leukemic activity and lack of in vivo toxicity were presented by AP/Tf-Drn/Lut NPs [91].

## 4. Aptamer Mediated Targeting of VEGF

Many anti-VEGF oligonucleotide aptamers capable of attaching with a high specificity and affinity to a particular biological target have also been created as prospective agents in anticancer therapies because of the significant clinical relevance of VEGF. By examining the analogous structures of the lead compounds, considerable research has focused on improving the discovered aptamers so as to boost target affinity and/or bioactivity.

Pegaptanib sodium is an oligonucleotide with a length of 28 nucleotides that ends in a pentyl amino linker. Two 20-kDa monomethoxy PEG units are covalently bonded to this linker through two amino groups on a lysine residue. As of right now, Pegaptanib (Macugen) is the only other intravitreal anti-VEGF medication licensed by the FDA for the treatment of neovascular age-related macular degeneration. It specifically binds to VEGF isoform 165 with a great affinity. By attaching to VEGF, it prevents neovascularization [92].

Specific aptamers can discriminate accurately between two protein isoforms because the molecular identification of their target is so precise. If one isoform is linked to an illness while the other is not, then this ability can be used therapeutically. The pro-angiogenic VEGF165a isoform of VEGF is most often expressed and recognized by Macugen. By functioning as a VEGF antagonist, when administered by intravitreal injection, Macugen binds explicitly to a pro-angiogenic variant of VEGF. It reduces vascularity and leakage, two neovascular age-related macular degeneration symptoms [93].

Proper diagnosis of aberrantly produced biomarkers, such as VEGF165, has been crucial in treating malignancies. Yet, recent advancements in aptamer-based diagnostics have made it possible to create tools that could replace the traditional methods of cancer biomarker evaluation. The analytical capabilities of aptamer-based diagnostic instruments (aptasensors) will determine how widely they are used in clinical settings [94].

**Figure 3:**
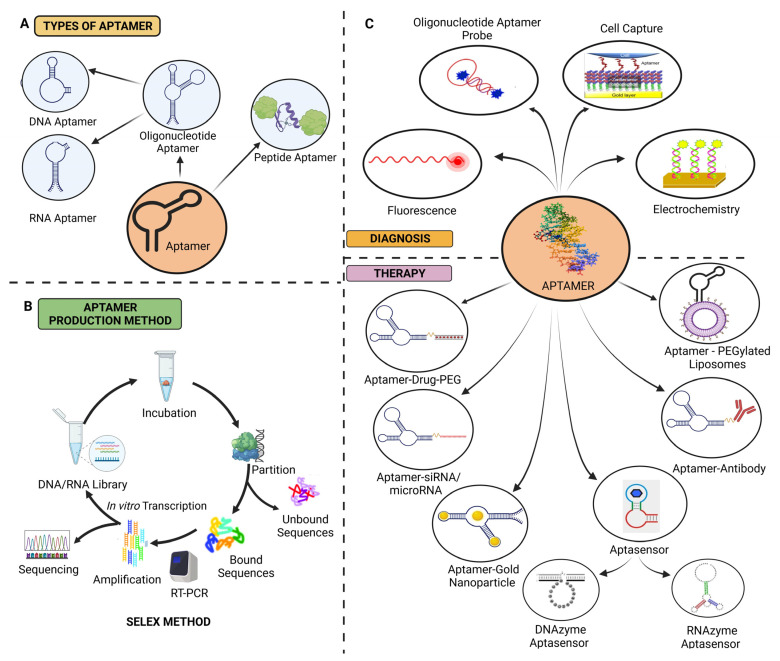
(**A**) Classification of aptamers, (**B**) schematic illustration of the SELEX technique, and (**C**) aptamers in ovarian cancer diagnosis and treatment [18,51,91].

VEGF receptor-1 (VEGFR-1), also known as fms-like tyrosine kinase-1 (Flt-1), and VEGF receptor-2 (VEGFR-2), also called as kinase insert domain-containing receptor (KDR), are the two major receptors for VEGF. VEGFR-1 signaling acts as a VEGF spoof receptor (Figure 3). The receptor is crucial for the activation of MMPs, haematopoiesis, and the invasion of monocytes along with other immune cells towards the TME [27,95].

VEGFR-2, on the other hand, is crucial for angiogenesis and vasculogenesis as it supports both processes through a variety of methods. Endothelial nitric oxide synthase (eNOS) and inducible nitric oxide synthase (iNOS) are both activated through the nitric oxide synthase (NOS) pathway as a result of VEGF binding to VEGFR-2. Nitric oxide (NO), one of the vasodilators released as a result of this signaling route, is released downstream, which increases vascular permeability. The activation of protein kinase B (PKB) through the binding of VEGF to VEGFR-2 can also stimulate phosphatidylinositol-3 kinase (PI3k), which in turn encourages EC survival, growth, and tube formation. In addition to the aforementioned actions, VEGF also stimulates angiogenesis by activating focal adhesion kinase (FAK), leading to cell migration via paxillin [96].

The affinity of VEGFR-2 for VEGF is around 10 times lower than that of VEGFR-1. The mitogenic effects of VEGF are mostly mediated by VEGFR-2 as it has a higher signaling activity than VEGFR-1. Additionally, VEGF-induced EC migration and vascular permeability are mediated by VEGFR-2, but VEGFR-1 only responds weakly or not at all [97].

Two DNA aptamers, Apt01 and Apt02, targeting VEGFR-1 and VEGFR-2, respectively, were chosen from libraries of 70-mer single-stranded oligodeoxynucleotides. Human umbilical vein endothelial cells were induced to form tubes by the aptamer Apt02 as a result of the differential in binding and nuclease stability. According to the findings, Apt02 could serve as a replacement for VEGF-A [98].

With an IC_50_ value of 49 pM, the Macugen aptamer binds to VEGF and prevents it from interacting with its receptors VEGFR-1 and VEGFR-2 [99]. A magnetic nanocrystal with an aptamer modification allowed for the exact identification of angiogenic vasculature using magnetic resonance (MR) imaging [100].

A thiolated hyaluronic acid (HA) polyethylene glycol (PEG) diacrylate hydrogel (tHA-PEGDA) containing immobilized RGD peptides for cell adhesion and anti-VEGF-R2 DNA aptamers was developed to promote angiogenesis without the need for external growth agents [101].

### 4.1. Aptamer-Based Biosensors Detecting VEGF

High-sensitivity detection of selected tumor markers allows for rapid detection and oncology treatment. A potential biomarker of tumor cells has recently been identified as VEGF165. Because of features such as cheap cost and quantitative analysis, the electrochemical aptasensor is a potential instrument for VEGF165 detection. Park et al. created a label-free electrochemical aptasensor based on carbon nanotube (CNT) and polyaniline composite (PANI) nanocomposites to identify VEGF165 as a tumor marker. The nanocomposite is put together with an immobilized VEGF165 aptamer to serve as a highly sensitive VEGF165 sensor. It is a very sensitive VEGF165 sensor; it displayed stable and broad linear detection ranges from 0.5 pg/mL to 1 g/mL, with a limit of detection of 0.4 pg/mL; it also displayed high selectivity when other proteins were present, good biological stability, and reproducibility after multiple measurement times following the dissociation process. After several measurement cycles, the manufactured aptasensor also showed high biological stability, reproducibility, and selectivity. Hence, it can be used as a non-invasive VEGF measurement [102].

**Figure 4:**
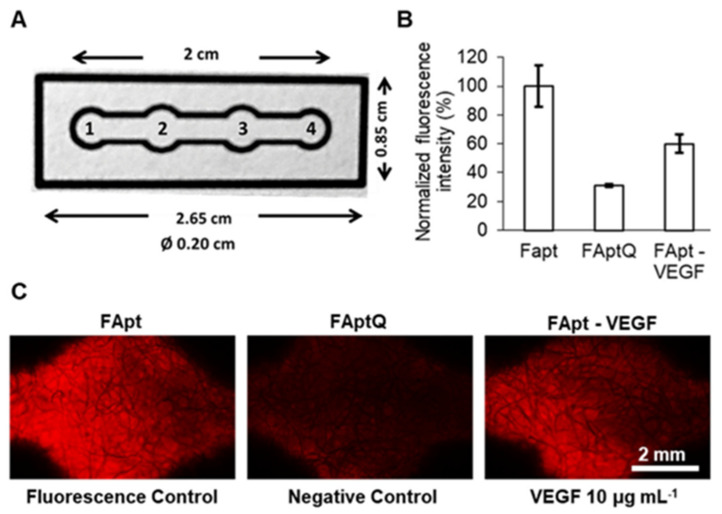
VEGF detection in μPAD: (**A**) images and information on μPAD. μPAD is divided into four zones: (1) the sample zone, (2) the VEGF detection zone, (3) the fluorescence control zone, and (4) the endpoint where the flow ceases. (**B**) A plot of the normalized fluorescence intensity obtained using the fluorescence control (FApt), negative control (FAptQ), and samples incubated with VEGF 10 μg mL^−1^ (FApt−VEGF) intensity. Error bars represent mean values ± standard deviation (*n* = 3). (**C**) Fluorescence in the detecting zone and fluorescence control zone incubated with and without VEGF, as seen via a microscope. Reproduced with permission from Ref. [103].

A three-part structural switching-signaling VEGF-specific sequenced aptamer was fabricated; upon VEGF identification, it displaced a quencher to provide a measurable fluorescence signal. In order to demonstrate this biosensor’s potential usage in cell biology research, it was incorporated into a microfluidic paper-based test device with VEGF detection and control. This biosensor was used to identify the VEGF released from a stem cell culture plate (Figure 4) [103].

In the angiogenesis of various malignancies, VEGF is a crucial biomarker for early cancer diagnosis; novel methods for VEGF detection that are quick, sensitive, and reliable are urgently needed. A sequence rich in guanine will produce a secondary structure called a guanine quadruplex (G-quadruplex), which is stabilized by Hoogsteen-type pairings between guanine bases. The G-quadruplex accumulation and assembly into Guanine threads (G-threads) are considerably promoted by the chaperone co-polymer PLL-g-Dex, allowing for efficient signal amplification. Through a stacking interaction known as π−π G-quadruplex can come together to form G-quadruplex nanowires (G-wires). As a result, self-assembled G-threads may be used to design optical and electrochemical biosensors as signal amplifiers. Han et al. looked into PLL-g-chaperone Dex’s abilities to help G-quadruplex fluorescent DNA biosensors accurately detect VEGF. The results show that the PLL-g-Dex molecular chaperone copolymer significantly promoted G-quadruplex accumulation and assembled into G-threads, providing efficient signal amplification [18].

Yuan et al. developed a brand new signal on−off−super on a sandwich-type aptamer sensor gold nanoparticles@Ti3C2Tx-Mxene (AuNPs@Ti3C2Tx-Mxene). An electrochemical signal was used to enhance the cleaved signal probes. They described a streamlined method of boosting electrochemical signals for detection. Under ideal circumstances, the aptamer sensor demonstrated great sensitivity, tolerable stability, and repeatability. They put out a cutting-edge method of CRISPR-Cas12a-based protein identification, which creates new opportunities for the diagnostic uses of diverse biomarkers [104].

The electrochemical aptasensor has a high affinity to recognize VEGF quickly. Based on recognizing aptamers and complex metallo-nanozyme particles that act as electron exchange centers and links between electrodes and captured DNA, they created VEGF aptasensors. Aptamers maintain their hairpin shape to avoid non-specific surface adsorption and reveal capture sequences when the target is absent. In contrast, the aptamers broke apart the hairpin structure to create room for VEGF and DNA to attach, increasing impedance. Electrochemical impedance spectroscopy (EIS) measures the electrochemical performance of the aptasensor. High specificity and repeatability were also demonstrated by the electrochemical aptasensor [105].

### 4.2. RNA and DNA Aptamers Detecting VEGF

Clinical diagnosis and scientific research both heavily rely on sensitive protein biomarker testing. Single-Molecule Recognition through Equilibrium Poisson Sampling (SiMREPS) is a technology that has recently become available for very sensitive protein, as well as other biomarkers, detection. The usefulness and benefit of the aptamer as a detection probe in SiMREPS were demonstrated using VEGF165 and IL8 as clinically pertinent biomarkers [106]. A therapeutic RNA aptamer called Macugen is used to target VEGF-165, the VEGF isoform most closely associated with angiogenesis. Cationic residues are found in all heparin-binding domains (HBD), and they interact with heparin’s anionic (carboxylate and sulfate) groups through electrostatic and hydrogen-bonding interactions to attach them. Macugen reportedly identifies HBD through a conformational selection mechanism. According to Kalathingal et al., Macugen detects HBD through a conformational selection process. In contrast, HBD recognizes Macugen through an induced-fit mechanism with significant conformational changes in Macugen and essentially without any changes in the HBD structure [107].

Nonaka et al. concentrated on the receptor-binding domain (RBD) of VEGF as a target epitope in order to produce an aptamer with an elevated affinity towards VEGF. Vap7, with K(d) values of 1.0 nM and 20 nM, respectively, bonded to the VEGF variants VEGF_121_ and VEGF_165_ after three rounds of screening. Furthermore, Vap7 demonstrated VEGF family specificity. Vap7 is predicted to fold to form a G-quadruplex structure using secondary structure calculations and circular dichroism. Only this portion of the aptamer sequence was present in the mutant aptamer that was produced. It had the sequence 5′-TGTGGGGGTGGACGGGCCGGGTAGA-3′ and seemed to take the form of a G-quadruplex. A second aptamer heterodimer was created by Nonaka et al., and it included aptamer (del5-1), which interacts with the heparin-binding domain of VEGF, associated with V7t1. With a K(d) value of 4.7 × 10^2^ pM, the resultant heterodimer has a considerable binding affinity for VEGF165 [108].

For an even greater affinity, two identical 3R02s were linked together by a 10-mer thymine linker to create a bivalent aptamer. With a K(d) value of 30 pM, the bivalent aptamer (3R02 Bi-valent) was bound to VEGF. Finally, a more accurate assay was created compared with the system using VEap121 by building a VEGF-detection method employing a VEGF antibody as the capture molecule, as well as monovalent 3R02 as the detection molecule. These findings suggest that in silico maturation may be a useful technique for increasing aptamer affinity for the development of sensitive detection systems [109].

In order to limit the conformational preferences of the G-quadruplex-forming VEGF-binding aptamer V7t1 to a single, distinct form, mutated nucleotides were inserted into its sequence. In a Na^+^-rich medium that mimics the extracellular environment where VEGF targeting should take place, Moccia et al. conducted an extensive biophysical characterization of V7t1 that used many approaches to examine the conformational behavior of the aptamer, as well as its binding to the protein. The findings show that, although annealed oligonucleotides are monomeric species, not-annealed V7t1 generates both monomeric and dimeric G-quadruplexes in the detection of high Na^+^ concentrations. Surprisingly, the sole dimeric aptamer successfully binds VEGF, demonstrating a stronger sensitivity for the protein than the monomeric form [110].

Napolitano et al. used the interaction of two aptamers forming G-quadruplexes V7t1 and 3R02 recognizing VEGF165 to develop effective cancer theranostic systems. Interestingly, both oligonucleotides were more effective at binding to VEGF165 when they adopted the dimeric G-quadruplexes that predominate over their monomeric versions in artificially healthy settings. These complexes have been successfully internalized into cells and co-localized with fluorescently labeled anti-VEGF-A antibodies, allowing for target recognition and detection. The assays demonstrate that the studied system is a viable tool for cancer theranostics [111].

Systematic examination of correlated signaling components in their natural context is fascinating for analyzing complex cellular signaling. This is still difficult because there are not many flexible and efficient solutions. By membrane-anchoring DNA processors, it is possible to suppress c-Met signaling more effectively and with more control. In addition, the DNA processor’s multitasking capabilities allowed for direct observation of the correlative VEGF secretion that was caused by controlling the activity of c-Met. Its modular DNA multitasking processor analyzed the coordination of cell surface receptors with associated components in living cells in detail by utilizing adaptable aptameric nucleic acids. As a result, it offers a potent chemical tool for applications in precision medicine and basic cytology research [112].

## 5. Aptamers Targeting VEGF in the Ovarian Cancer Therapy

VEGF, which is involved in cell development, proliferation, and angiogenesis, may contribute to the etiology of cancer. The most effective method for detecting early-stage OC without metastasis is to detect VEGF.

To identify VEGF in OC, an oligonucleotide-aptamer-based colorimetric assay is utilized. The target aptamer is present when the unmodified gold nanoparticle (GNP) is employed in the colorimetric test. When there is a high salt concentration present, such as sodium chloride (NaCl), free GNP then becomes blue in hue. Even at high salt concentrations, GNP retains its natural red color when the aptamer binds to the GNP’s surface in the absence of VEGF. With this test, the VEGF detection limit is 185 pM. Less than 500 ng/mL of VEGF is the typical range. The photothermal treatment also employs GNP-conjugated aptamers. The intensity of fluorescence will alter with aptamer and VEGF binding [88,113,114].

Gold magnetic (Au-Fe_3_O_4_) nanoparticles with dual surfaces that resemble dumbbells have been created as clever photo-controlled drug carriers for precise aptamer distribution. Electrostatic absorption was used to build DNA aptamers that target VEGF and Au-Fe_3_O_4_ NP, and Apt-Au-Fe_3_O_4_ NP has been shown to selectively attach to SKOV-3 OC cells. Aptamers are considerably released in cells when exposed to plasma resonance light (605 nm), which improves the prevention of tumor cell growth in vitro [18].

Ji et al. developed an alkalinity-dependent fluorescence enhancement-based method to measure VEGF165 in human blood using hairpin DNA silver nanoclusters (hDNA-AgNC) and oxidized carbon nanoparticles (CNP). A hairpin loop containing AgNCs, a hairpin rod with a stable structure, and a terminal aptamer 1 constitute the hDNA-AgNCs aptamer detection probe (with aptamer 2 to recognize the target). hDNA-AgNCs showed approximately three times more intensity for fluorescence compared with that of single-stranded DNA AgNCs (ssDNA-AgNC). The number of bases in the stem and hairpin loop greatly affected the fluorescence. As a result, the fluorescence was recovered, and the hDNA-AgNCs were no longer close to the oxidized surface of CNP, thereby reducing the efficiency of FRET. The aptasensing probe has a detection limit of 14 pM and can only assess VEGF165 under optimal conditions. Discovery provides a sensitive, label-free method to detect VEGF165 in human serum using a DNA aptamer [115].

Dong et al. worked on a sandwich-type colorimetric microplate detector using a VEGF-specific biotinylated DNA aptamer. The recombinant VEGF protein was fixed in microplate wells in the first phase, then the biotinylated aptamer was added. To visualize the aptamer−VEGF complex, streptavidin and horseradish peroxidase were conjugated. A subsequent washout removed any surface-unbound aptamers, and the colorimetric signal diminished as the level of VEGF increased. The devised assay had a detection limit of 0.3 pM in the buffer solution. VEGF in the human serum can be detected using this aptasensor. The acquired results were consistent with the results of the standard chemiluminescent ELISA technique. Interestingly, the developed aptasensor provided quick and easy detection of VEGF in blood [116].

Aptamers are sometimes denoted as “chemical antibodies” as they share the same target selectivity as conventional antibodies and have a similar capacity to attach to biomarkers through structural recognition. Additionally, aptamers’ interactions with their molecular targets have binding specificities and potencies that are occasionally much greater than those of antibodies [93].

Aptamers may be employed in tumor-targeted treatment to overcome multidrug resistance because they may effectively distribute aptamer-mediated nanoparticles into cancer cells of the ovary and overcome multidrug resistance brought on by VEGF. Aptamers made with anti-VEGF antibodies have the potential to treat ovarian cancer by blocking blood vessels. Targeting VEGF, DNA aptamers are dramatically released in cells and improve the in vitro suppression of tumor cell growth (Table 2). Aptamers and nanomaterials work together to increase the rate of early VEGF detection and the effectiveness of targeted ovarian cancer therapy [18].

Aptamer-based technologies remain in their infancy, and further study is required to guarantee their general acceptance. Despite several clinical studies, aptamer therapeutic uses have been sluggish to gain popularity. The US Food and Drug Administration (USFDA) has clinically approved only Pegaptanib (Macugen). One of the most crucial difficulties with anti-VEGF aptamers is biocompatibility. The standard technology of aptamers is advancing while their production cost remains expensive compared with traditional antibodies [105].

The greater lactic acid (LA) content (2.2 mM) in serum samples can still cause a small amount of interference with the aptasensing probe’s ability to detect VEGF165. The serum samples are first processed with NAD+ by lactate dehydrogenase (LDH) (catalyzing) to remove LA, and then the procedure is used to determine the results. In addition to these, the assay method may be expanded to identify numerous cancer markers by swapping out various target aptamers coupled at the 5′-end of hDNA-AgNCs [114].

Although they frequently rely on aptamer/antibody pairings, colorimetric aptasensors offer particularly high sensitivity and specificity for detecting VEGF in ovarian cancer. The created aptasensor exhibited a great degree of sensitivity, but a significant nonspecific signal showed up due to spontaneous quadruplex formation in the absence of a target. Only in an abundance of VEGF, which dramatically decreased the nonspecific signal, did the active quadruplex structure arise when the aptamer was split into two distinct oligonucleotides. The threshold of detection limit was consequently decreased to 2.6 nM [116].

VEGF plays a critical role in angiogenesis and is involved in various physiological and pathological processes, including cancer development and progression. In the context of cancer, VEGF promotes the growth of blood vessels around tumors, enabling their sustained growth and providing nutrients for metastasis. Aptamers can bind to specific target molecules with a high affinity and selectivity. They have gained attention as potential diagnostic and therapeutic agents due to their ability to recognize and interact with specific molecular targets, such as VEGF.

To specifically and accurately make and use VEGF aptamers for diagnosing and treating OC, a few steps can be followed:

(i) Selection of VEGF-specific aptamers: The process typically involves an in vitro selection method called SELEX, in which a random library of aptamers is generated, and multiple rounds of selection and amplification are performed to enrich the aptamers that bind specifically to VEGF [18].
(ii) Validation of aptamer binding: After several rounds of SELEX, the selected aptamers are tested for their binding affinity and specificity towards VEGF. This can be done using techniques such as surface plasmon resonance (SPR) or fluorescence-based assays.
(iii) Diagnostic applications: Once VEGF-specific aptamers are identified, they can be utilized for diagnostic purposes in OC. For example, aptamers can be conjugated with fluorescent or radioactive labels to develop imaging probes that specifically target VEGF-expressing tumor cells. These probes can help visualize and detect the presence of OC lesions.
(iv) Therapeutic applications: VEGF aptamers can also be explored as therapeutic agents. By binding to VEGF, aptamers can interfere with its activity and inhibit angiogenesis, thus limiting tumor growth and metastasis. Additionally, aptamers can be engineered to deliver therapeutic payloads, such as drugs or siRNAs, specifically to VEGF-expressing cancer cells.

It is important to note that while VEGF is a common target in many types of cancer, including OC, the specificity of aptamers can be fine-tuned during the SELEX process. By using appropriate selection conditions and controls, it is possible to isolate aptamers that specifically recognize VEGF and minimize their cross reactivity with other proteins or different types of cancer. However, it is crucial to conduct extensive experimental validation to ensure the specificity and efficacy of VEGF aptamers in diagnosing and treating OC specifically. The development and optimization of aptamers for clinical use require rigorous testing, including preclinical studies and eventually clinical trials, in order to demonstrate their safety and efficacy in OC patients [18,51,52,53,54].

Despite all of the difficulties, aptamer applications in anti-VEGF therapy for OC treatment are progressing in the path of new therapeutic development. The effects and side effects of aptamers in the anti-VEGF therapy of OC are represented in Table 2.

## 6. Conclusions and Future Prospects

Ovarian cancer (OC) is the second greatest cause of cancer-associated deaths in females, with most cases diagnosed as advanced metastatic disease when treatment options are limited. To create novel treatments, it is crucial to thoroughly investigate the characteristics of cancer cells, one of which is their resistance to apoptosis, which unquestionably contributes significantly to the growth and spread of tumors. In particular, the up-regulation of antiapoptotic genes, such as certain members of the Bcl-2 family of proteins and members of the inhibitor of apoptosis (IAP) family of proteins, is associated with the capacity of cancer cells to avoid apoptosis. Recent research has demonstrated that the SMAC/DIABLO mimic LBW242 induces caspase-8 activation in OC cells [117].

There is an urgent need to develop powerful identification tools for OC-related biomarkers, such as VEGF, to achieve early diagnosis and precise treatment. VEGF is a key biomarker of angiogenesis in several cancers, including OC. The development of aptamer-anti-VEGF strategies is auspicious. It is preferable to utilize aptamers that create hairpin-forming aptamers, such as Pegaptanib or G-quadruplexes, such as Vap7, V7t1, 3R02, and VEap121, and its equivalents. Pegaptanib is a 28-nucleotide RNA aptamer that is selective for the VEGF165 isoform. In order to create VEGF-targeted anticancer treatments, various teams have thoroughly characterized new covalent V7t1 dimers. The bisV7t1TEG2D analog had a non-nucleotide linker that introduced a 3′-3′ inversion from the polarity site in the center of its chain and supported parallel G4 conformation. This reduced the overall polymorphism of V7t1 [109,110,111,112].

Our knowledge of angiogenesis and vasculogenesis has been fundamentally altered by the discovery of VEGF and its essential biological functions. This research has also added a new tool to the anticancer toolbox: the targeted suppression of this protein. The discovery of VEGF’s molecular regulation and the creation of innovative treatment strategies that either directly or indirectly target VEGF, serves as an amazing case study demonstrating the importance of fundamental research for directing innovation and translational medicine.

Parallel to this, the precise and quick assessment of circulating VEGF levels is crucial for cancer diagnosis, prognosis, monitoring, and management. In fact, pharmacological combination therapy can significantly improve existing anticancer treatments because of the biological intricacy of VEGF signaling. Aptamers can open new avenues by targeting VEGF in OC treatment. Right now, there are very few studies regarding the application of aptamers targeting VEGF in OC cell lines, and we hope that old approved aptamer drugs and ongoing research will open new horizons.

## Figures and Tables

**Table 1:** Aptamers detecting VEGF as potential biomarkers for the clinical diagnosis of OC together with common biomarkers of OC.

Aptamer Type	Biomarkers of Ovarian Cancer	Diagnostic Rate	Reference
DNA aptamer	VEGF	0.185 nM	[85]
DNA aptamer	CA125	0.05 U/mL	[84]
DNA aptamer	HE4	13 nM	[74]
Tx-01 aptamer	HSP70	200 μm	[75]
Amine functionalized aptamer	MUC-1	0.8 nM	[82]
Aptasensor	CEA	0.5 ng/mL	[83]

**Table 2:** The effects and side-effects of aptamers in the anti-VEGF treatments of OC.

Aptamer-Based Anti-VEGF Treatments	Effects	Side Effects	Reference
Aptamer-modified magnetic nanocrystal	Accurate identification of angiogenic vessels	No immunogenic responses in vivo	[100]
Bifunctional thiolated hyaluronic acid−polyethylene glycol diacrylate hydrogels	Despite exogenous growth agents, stimulating angiogenesis	Boost cell survival, encourage cell movement, and accelerate angiogenesis	[101]
Alkalinity-dependent fluorescence enhancement-based treatment	High sensitivity and selectivity	Significant cytotoxicity and improved anti-tumor efficiency	[114]
Colorimetric assay	High sensitivity	Lower detection limit	[115]
Sandwich-type colorimetric microplate detector	Quick and easy detection of VEGF in blood	Higher anti-tumor efficiency	[116]

## Data Availability

Not applicable.
